# Phylogenetic patterns in regional flea assemblages from 6 biogeographic realms: strong links between flea and host phylogenetic turnovers and weak effects of phylogenetic originality on host specificity

**DOI:** 10.1017/S003118202300015X

**Published:** 2023-04

**Authors:** Boris R. Krasnov, Georgy I. Shenbrot, Irina S. Khokhlova

**Affiliations:** 1 Mitrani Department of Desert Ecology, Swiss Institute for Dryland Environmental and Energy Research, Jacob Blaustein Institutes for Desert Research, Ben-Gurion University of the Negev, Sede Boqer Campus, Midreshet Ben-Gurion, Israel; 2 French Associates Institute for Agriculture and Biotechnology of Drylands, Jacob Blaustein Institutes for Desert Research, Ben-Gurion University of the Negev, Sede Boqer Campus, Midreshet Ben-Gurion, Israel

**Keywords:** Biogeographic realms, environment, fleas, hosts, phylogenetic originality, phylogenetic turnover

## Abstract

We investigated phylogenetic patterns in flea assemblages from 80 regions in 6 biogeographic realms and asked whether (a) flea phylogenetic turnover is driven by host phylogenetic turnover, environmental dissimilarity or geographic distance; (b) the relative importance of these drivers differs between realms; and (c) the environmental drivers of flea phylogenetic turnover are similar to those of host phylogenetic turnover. We also asked whether the phylogenetic originality of a flea species correlates with the degree of its host specificity and whether the phylogenetic originality of a host species correlates with the diversity of its flea assemblages. We found that host phylogenetic turnover was the best predictor of flea phylogenetic turnover in all realms, whereas the effect of the environment was weaker. Environmental predictors of flea phylogenetic turnover differed between realms. The importance of spatial distances as a predictor of the phylogenetic dissimilarity between regional assemblages varied between realms. The responses of host turnover differed from those of fleas. In 4 of the 6 realms, geographic distances were substantially better predictors of host phylogenetic turnover than environmental gradients. We also found no general relationship between flea phylogenetic originality and its host specificity in terms of either host species richness or host phylogenetic diversity. We conclude that flea phylogenetic turnover is determined mainly by the phylogenetic turnover of their hosts rather than by environmental gradients. Phylogenetic patterns in fleas are manifested at the level of regional assemblages rather than at the level of individual species.

## Graphical abstract

**Figure:**
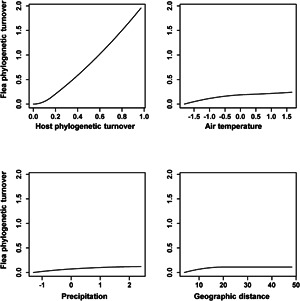

## Introduction

A link between species diversity and habitat diversity was first reported more than a half-century ago (MacArthur, 1958, 1964) and was further supported for a variety of plant and animal species (Lawton, 1983; Rosenzweig, 1995; Tews *et al*., 2004). Given that hosts represent the ultimate habitats for a majority of parasite taxa, it is thus not surprising that studies of the effects of host diversity on parasite diversity have reported strong positive associations between them (Watters, 1992; Krasnov *et al*., 2004*a*; Thieltges *et al*., 2011; Kamiya *et al*., 2014). However, parasite diversity is determined not only by host diversity, but also by environmental factors (e.g. Adlard *et al*., 2015). In other words, parasite species composition results from an interplay between host species composition and environment. This is especially true for ectoparasites because they are affected by both hosts and the external off-host environment (Marshall, 1981). Obviously, compositional diversity is not the only component of species diversity. Phylogenetic diversity is no less important because it represents an evolutionary measure of biodiversity that may or may not be reflected by compositional diversity (Miller *et al*., 2018). Although phylogenetic methods are now broadly applied in parasitological studies (Bass *et al*., 2015; Selbach *et al*., 2019), investigations of the patterns of phylogenetic diversity in parasites are still more scarce than those of free-living taxa (but see Clark, 2018; Krasnov *et al*., 2019*a*, 2019*b*; Llopis-Belenguer *et al*., 2020).

One of the main questions asked in studies on parasite diversity is whether it is mainly driven by host diversity or environmental factors. The majority of studies on compositional parasite diversity have suggested that the effect of host diversity was more important than that of environmental variation. For example, the diversity of helminth parasites of amphibians, at the global scale, was found to be affected by both precipitation seasonality and host richness, with the effect of the latter being stronger (Martins *et al*., 2021). The meta-analysis of Kamiya *et al*. (2014) convincingly demonstrated that parasite species richness is strongly correlated with that of their hosts. A study on fleas in northern and central Eurasia suggested that the drivers of flea community composition were scale-dependent, with host composition playing the main role on the continental scale, whereas the effect of environment was more important on the regional scale (Krasnov *et al*., 2015). In contrast to compositional diversity, the drivers of parasites' phylogenetic diversity have been poorly studied. Nevertheless, it appeared that the relative effects of host phylogenetic diversity and environment on parasite diversity could vary between geographic regions, even in the same parasite taxon. Among the drivers of the phylogenetic diversity of regional flea assemblages within each of 4 biogeographic realms (the Afrotropics, the Nearctic, the Neotropics and the Palaearctic), host phylogenetic diversity played the most important role in the Palaearctic only, whereas the effect of environment was weak, although the opposite was true for the Afrotropics and the Nearctic (Krasnov *et al*., 2019*a*). The latter study dealt with phylogenetic alpha-diversity. Phylogenetic beta-diversity (i.e. dissimilarity in phylogenetic composition between communities) may respond to factors other than compositional beta-diversity. Furthermore, it is unknown whether the drivers of phylogenetic beta-diversity of the same parasite taxon vary between geographic regions or whether these drivers are geographically invariant, as is the case with parasite compositional beta-diversity. In fact, dissimilarity in host species composition, across regions in all 4 aforementioned biogeographic realms, was the most important factor affecting the compositional dissimilarity of flea assemblages, whereas environmental effects were weaker (Krasnov *et al*., 2020). A similar pattern has been reported for chiropterans and their bat fly parasites (Eriksson *et al*., 2019). The results of 2 studies on the phylogenetic beta-diversity of fleas were obtained at a relatively small scale (Krasnov *et al*., 2019*b*; Maestri *et al*., 2020) and do not allow answering the question about the geographic variation of predictors of parasite phylogenetic turnover at a large scale.

Elucidating the relationships between the beta-diversities (whether compositional or phylogenetic) of 2 different taxa or between beta-diversity and environment is essentially a test of the relationships between either species (or phylogenetic lineages) turnover of taxon A (e.g. parasites) and taxon B (e.g. hosts) or species (or phylogenetic lineages) turnover and environmental dissimilarity. The drivers of community compositional/phylogenetic turnover should be analysed with an appropriate, non-linear analytical method represented, for example, by generalized dissimilarity modelling (GDM) (Ferrier *et al*., 2007; Mokany *et al*., 2022). This is because of 2 main problems associated with any linear analysis of pairwise metrics of dissimilarity/turnover, namely that (a) the range of any dissimilarity index varies from 0 to 1 only, and (b) the rate of species (or phylogenetic lineages) compositional change along a gradient is not necessarily constant, so the model has to take into account the curvilinear relationship between dissimilarity in species/phylogenetic composition and environmental dissimilarity or geographic distance. The GDM resolves both these problems and, moreover, can incorporate various biotic and abiotic predictors into a single model. The GDM has been successfully, albeit rarely, applied in studies of parasite species turnover and has proven to be a promising tool for revealing drivers of parasite diversity (Maestri *et al*., 2017, 2020; Eriksson *et al*., 2019; Krasnov *et al*., 2020; McNew *et al*., 2021). Phylogenetic generalized dissimilarity modelling (phyloGDM) (Ferrier *et al*., 2007) represents an extension of the traditional GDM aimed at modelling spatial patterns of phylogenetic, rather than compositional, turnover (Ferrier *et al*., 2007; Rosauer *et al*., 2013).

Another phylogenetic pattern in parasite–host associations is related to the phylogenetic originality of either a parasite or a host. A species is considered original if it has only a few close relatives and/or if its branch in the phylogenetic tree is long (i.e. it supposedly evolved quickly) (May, 1990; Vane-Wright *et al*., 1991; Faith, 1992; Pavoine *et al*., 2005; Pavoine and Izsak, 2014). Phylogenetically original species are highly interesting from the conservation point of view because of their important roles within communities and ecosystems and increased probability of extinction (e.g. Isaac *et al*., 2007, 2012; but see Pavoine *et al*., 2017). Relationships between a species’ degree of phylogenetic originality and its ecological features are poorly known. Nevertheless, the results of the few available studies suggest that phylogenetically original species tend to have restricted geographic ranges (Cadotte and Davies, 2010; Veron *et al*., 2021). Taking into account one of the most persistent macroecological patterns, namely the negative relationship between geographic range size and niche breadth (Brown, 1984), it can be hypothesized that phylogenetic originals are likely ecological specialists. This has never been specifically tested either in free-living species or in parasites. From a parasite perspective, niche specialization can be reflected in a high degree of host specificity, both structural and phylogenetic (sensu Poulin *et al*., 2011; see Krasnov *et al*., 2005 for fleas). From a host perspective, if phylogenetically original hosts are also those with a restricted geographic range, then a decrease in the diversity of the parasite assemblages they harbour with an increase in the degree of their phylogenetic originality can be expected because of the negative relationships between a host's geographic range size and the diversity of its parasite assemblage (Feliu *et al*., 1997; Krasnov *et al*., 2004*b*).

Here, we studied phylogenetic patterns of regional flea assemblages in 6 biogeographic realms (the Afrotropics, the Australasia, the Indomalaya, the Nearctic, the Neotropics and the Palaearctic). The aims of this study were 2-fold. First and similar to earlier studies of flea compositional dissimilarity (e.g. Krasnov *et al*., 2019*b*), we asked (a) whether flea phylogenetic turnover is mainly driven by host phylogenetic turnover or environmental dissimilarity or alternatively by the geographic distance between flea/host assemblages and (b) whether the patterns of the relationships between flea phylogenetic turnover, host phylogenetic turnover, environmental dissimilarity and geographic distance differ between biogeographic realms due to differences in flea and host evolutionary histories in these realms (Traub, 1980; Medvedev, 2005). To assess the relationships between flea and host phylogenetic turnover, environmental dissimilarity and geographic distances, we applied phyloGDMs. In addition, to understand whether spatial patterns and environmental drivers of flea phylogenetic turnover are similar to those of host phylogenetic turnover (Maestri *et al*., 2017), we applied phyloGDMs to host phylogenetic turnover as affected by environmental dissimilarity and geographic distances. Second, we asked whether the phylogenetic originality of a flea species correlated with the degree of its host specificity in terms of either size (i.e. the number of host species) or the phylogenetic diversity of its host spectrum. We also asked whether the phylogenetic originality of a host species correlated with the number of flea species it harbours or their phylogenetic diversity.

## Materials and methods

### Data on fleas, hosts and their interactions

Data on fleas parasitic on small mammalian hosts were taken from a variety of published surveys that reported identities of flea species collected from a given host species [Monotremata, Dasyuromorphia, Didelphimorphia (except large opossums), Paramelemorphia, Notoryctemorphia, Diprotodontia (except Vombatiformes), Macropodiformes (except large kangaroos), Paucituberculata, Macroscelidea, Eulipotyphla, Rodentia and ochotonid Lagomorpha] from 15 regions of the Afrotropics, 8 regions of the Australasia, 10 regions of the Indomalaya, 23 regions of the Nearctic, 17 regions of the Neotropics and 36 regions of the Palaearctic (see lists of regions, maps and references in Krasnov *et al*., 2022*a*). Similarly to our earlier study (Krasnov *et al*., 2022*b*), whenever possible, we relied on the information in the original sources and did not include in the analyses those findings of certain fleas on certain hosts that the author(s) of the original publications considered to be accidental. Ubiquitous host (*Rattus rattus*, *Rattus norvegicus* and *Mus musculus*) and flea (*Xenopsylla cheopis*, *Xenopsylla brasiliensis*, *Nosopsyllus fasciatus*, *Nosopsyllus londoniensis* and *Leptopsylla segnis*) species, as well as introduced host species (e.g. *Ondatra zibethicus* in the Palaearctic), were also excluded from the analyses. In total, we used data on 213 flea and 196 host species in the Afrotropics, 147 flea and 140 host species in the Australasia, 236 flea and 147 host species in the Indomalaya, 254 flea and 217 host species in the Nearctic, 200 flea and 160 host species in the Neotropics and 338 flea and 209 host species in the Palaearctic.

### Phylogenies

Phylogenetic trees for fleas and hosts were constructed for each realm separately because flea faunas, as well as the faunas of flea-harbouring hosts, have been shown to form clear clusters according to the biogeographic realms in which they occur (Krasnov *et al*., 2022*a*). For fleas, we used, as a backbone, the most recent and comprehensive (and 1 of only 2 available) molecular phylogenetic tree (Zhu *et al*., 2015). This tree comprised the majority of flea genera from our datasets, whereas this was not the case for the majority of species. The topology of the remaining genera and species was established based on either their morphologically derived taxonomic positions (Hadfield *et al*., 2014) or the molecular/morphological phylogenies carried out for some genera (see references in Krasnov *et al*., 2022*b*). No information on branch length for ca. 80% of species was available. Consequently, we arbitrarily assigned all branch lengths to a length of 1 and arbitrarily ultrametrized the resultant tree using the option ‘arbitrarily ultrametrize’ in the Mesquite modular system for evolutionary analysis (Maddison and Maddison, 2021). Polytomies (9 clades in the Indomalaya, 3 clades in the Nearctic and 11 clades in the Palaearctic) were resolved using the function ‘fix.poly’ of the package ‘RRphylo’ (Castiglione *et al*., 2020) of the R programming language for statistical computing (R Core Team, 2022).

Host phylogenetic trees were taken as subsets from the 10 000 species-level birth–death tip-dated completed trees for 5911 mammal species of Upham *et al*. (2019). We built a consensus tree for each realm from 1000 random trees of Upham *et al*. (2019) using the function ‘consensus.edge’ of the R package ‘phytools’ (Revell, 2012), ultrametrized this tree using the function ‘force.ultrametric’ (with option method = ‘extend’) of the ‘phytools’, and resolved the polytomies using the function ‘fix.poly’ of the ‘RRphylo’.

### Environmental data

The latitudinal and longitudinal positions of the centres of the regions were determined using ArcGIS 10.6 based on the information from the original source. Environmental variables for each region included (a) seasonal normalized difference vegetation indices (NDVI) (reflecting the amount of green vegetation in boreal/austral spring, summer, autumn and winter); (b) mean, maximum and minimum air temperature; and (c) seasonal precipitation (for boreal/austral spring, summer, autumn and winter). Environmental data were averaged over a region across 30 arc-second grids. NDVI data were obtained from the database PROBA-V S10 TOC NDVI 1KM (http://www.eovoc.spacebel.be). Air temperature and precipitation variables were extracted from the WORLDCLIM (BIOCLIM) 2.0 package (Fick and Hijmans, 2017). Then, we ran principal component analyses of each category of environmental variables and substituted their original values with the scores of the first principal component. This resulted in 3 composite environmental variables, namely (a) a vegetation variable (based on the NDVI) that reflected the amount of green vegetation, (b) an air temperature variable and (c) a precipitation variable. In each realm, these new variables explained from 72 to 97% of the variation in NDVI, from 74 to 97% of the variation in air temperature and from 71 to 92% of the variation in precipitation (see Supplementary Table S1). In all realms, the vegetation, air temperature and precipitation variables reflected an increase in the respective raw variables, except in the Indomalaya, where the principal component of precipitation variables corresponded to a decrease in the amount of summer rainfall and an increase in rainfall in the remaining seasons (Supplementary Table S1).

### Data analyses: phyloGDM

Measures of phylogenetic dissimilarities among sites represent traditional dissimilarity indices where species are replaced by evolutionary units (Ferrier *et al*., 2007; Nipperess *et al*., 2010; Pavoine, 2016). For each realm, we constructed a presence/absence matrix for either flea or host species per each region. Then, we calculated flea or host phylogenetic dissimilarity matrices for each realm using the command ‘evodiss_family’ of the R package ‘adiv’ (Pavoine, 2020) with option ‘method = 7’, that is, using coefficient S12 of Gower and Legendre (1986) based on Ochiai (1957). We chose to use this coefficient because it is calculated for incidence rather than abundance data, whereas the abundances of either flea or host species were not available in the majority of data sources. PhyloGDMs were carried out separately for each realm using the R package ‘gdm’ (Fitzpatrick *et al*., 2022). First, we ran phyloGDMs to reveal how between-region phylogenetic dissimilarity in flea assemblages was affected by host phylogenetic dissimilarity, environmental dissimilarity and geographic distances. Then, we ran phyloGDMs on host phylogenetic dissimilarity as affected by environmental dissimilarity and geographic distances.

### Data analyses: phylogenetic originality and phylogenetic diversity

To test (a) whether the phylogenetic originality of a flea species affected its level of host specificity in terms of the size and phylogenetic diversity of a host spectrum and (b) whether the phylogenetic originality of a host species affected the species richness and phylogenetic diversity of its flea assemblages, we calculated the measures of the phylogenetic originality of a flea or a host species and the phylogenetic diversity of a host spectrum or a flea assemblage separately for each realm. The phylogenetic originality of a flea or a host was calculated as 2Hb index (a vector that maximizes the ^2^*H* index of Pavoine and Izsak, 2014) using the function ‘distinctUltra’ with the option ‘method = 2Hb’ of the ‘adiv’ package. ^2^*H* represents an application of the quadratic entropy (QE) of Rao (1982) modified by Ricotta and Szeidl (2009) and developed by Pavoine *et al*. (2005) and Pavoine and Izsak (2014), where the QE is equal to the expected dissimilarity between 2 entities randomly selected using a replacement (see mathematical details in Pavoine and Izsak, 2014). Pavoine and Izsak (2014) demonstrated that a maximizing vector for ^2^*H* can be used as a measure of a given species’ phylogenetic originality.

The sizes of a flea's host spectrum and the species richness of a host's flea assemblage were calculated as mere numbers of species. Mean pairwise distance (MPD) and mean nearest-taxon distance (MNTD) (Tucker *et al*., 2017) were used as metrics of the phylogenetic diversity of either a flea's host spectrum or a host's flea assemblage. Both metrics measure the phylogenetic dispersion of a community. MPD is the average phylogenetic distance between all species, whereas MNTD measures the average phylogenetic distance between the nearest-neighbouring species. The standardized effect sizes of the MPD and MNTD (SES_MPD_ and SES_MNTD_, respectively) for either host spectra or flea assemblages were obtained by comparing observed phylogenetic relatedness to phylogenetic relatedness expected under the null model of randomization of either a host spectrum or a flea assemblage constructed using the ‘independent swap’ algorithm (Gotelli and Entsminger, 2001). This algorithm maintains species occurrence frequency and species richness in either a host spectrum or a flea assemblage. Calculations were done using the functions ‘ses.mpd’ and ‘ses.mntd’, respectively, of the R package ‘picante’ (Kembel *et al*., 2010).

The relationships between a flea species’ or a host species’ originality (an independent variable) and either the size/species richness of a host spectrum or a flea assemblage or its phylogenetic diversity (a dependent variable) were analysed using generalized linear models. Prior to the analyses, all variables were normalized from 0 to 1.

## Results

### Phylogenetic turnover

**Fig. 1.**
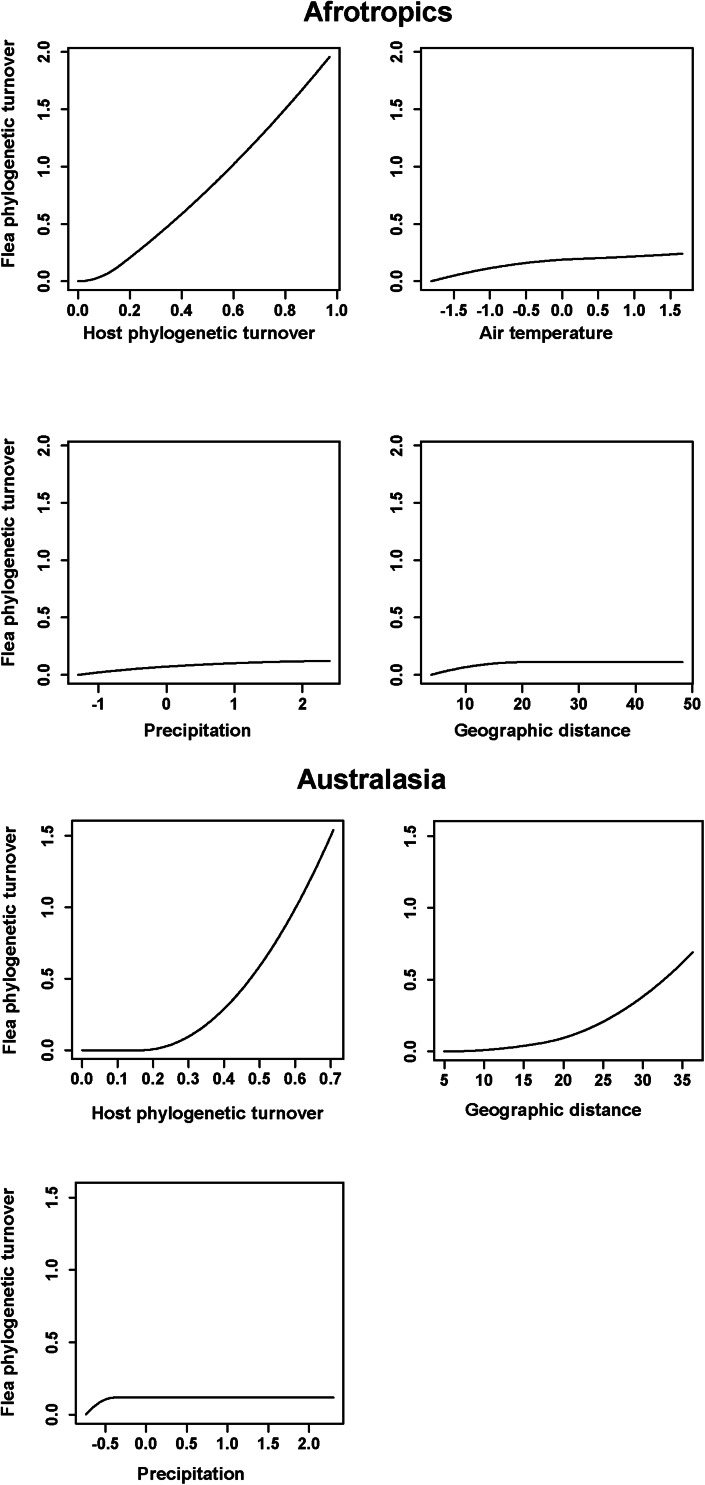
Generalized dissimilarity model-fitted I-splines (partial regression fits) of host phylogenetic turnover, environmental variables and geographic distance as predictors of flea phylogenetic turnover across the Afrotropics and the Australasia. The steeper slope of the transformed relationship on a given section of the gradient indicates a greater rate of turnover.

**Fig. 2.**
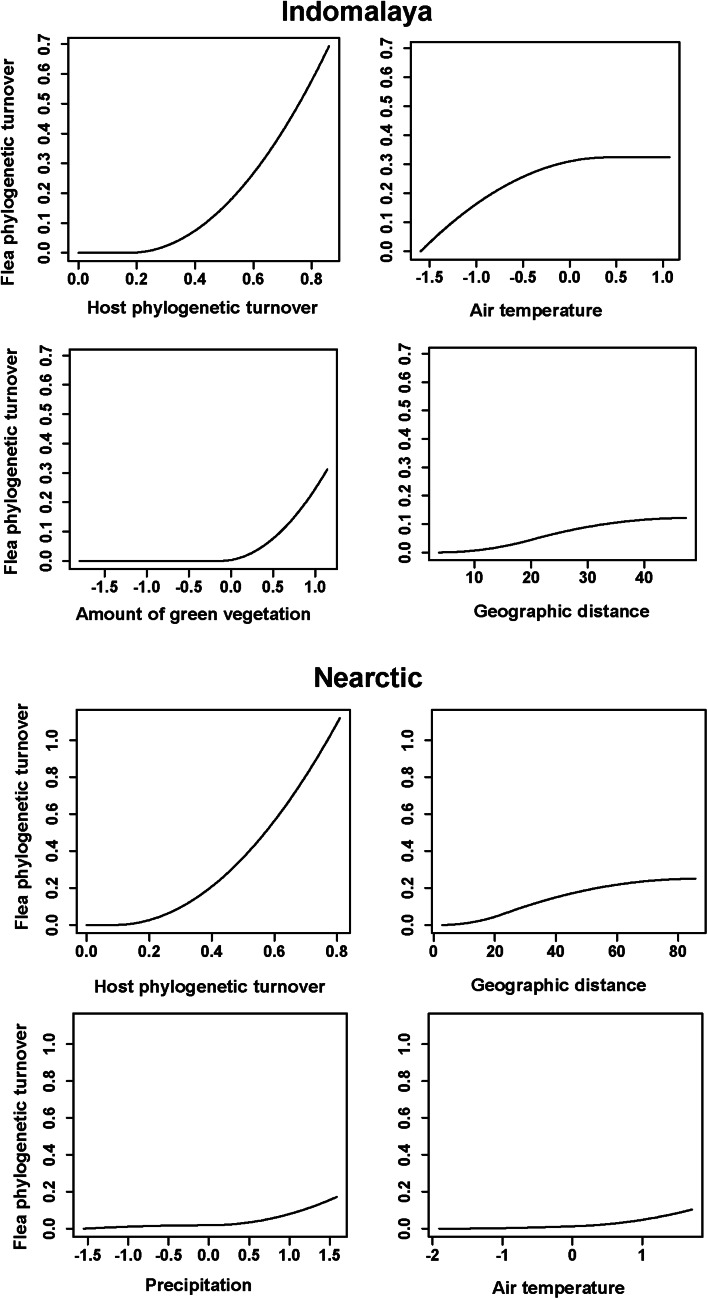
Generalized dissimilarity model-fitted I-splines (partial regression fits) of host phylogenetic turnover, environmental variables and geographic distance as predictors of flea phylogenetic turnover across the Indomalaya and the Nearctic. The steeper slope of the transformed relationship on a given section of the gradient indicates a greater rate of turnover.

**Fig. 3.**
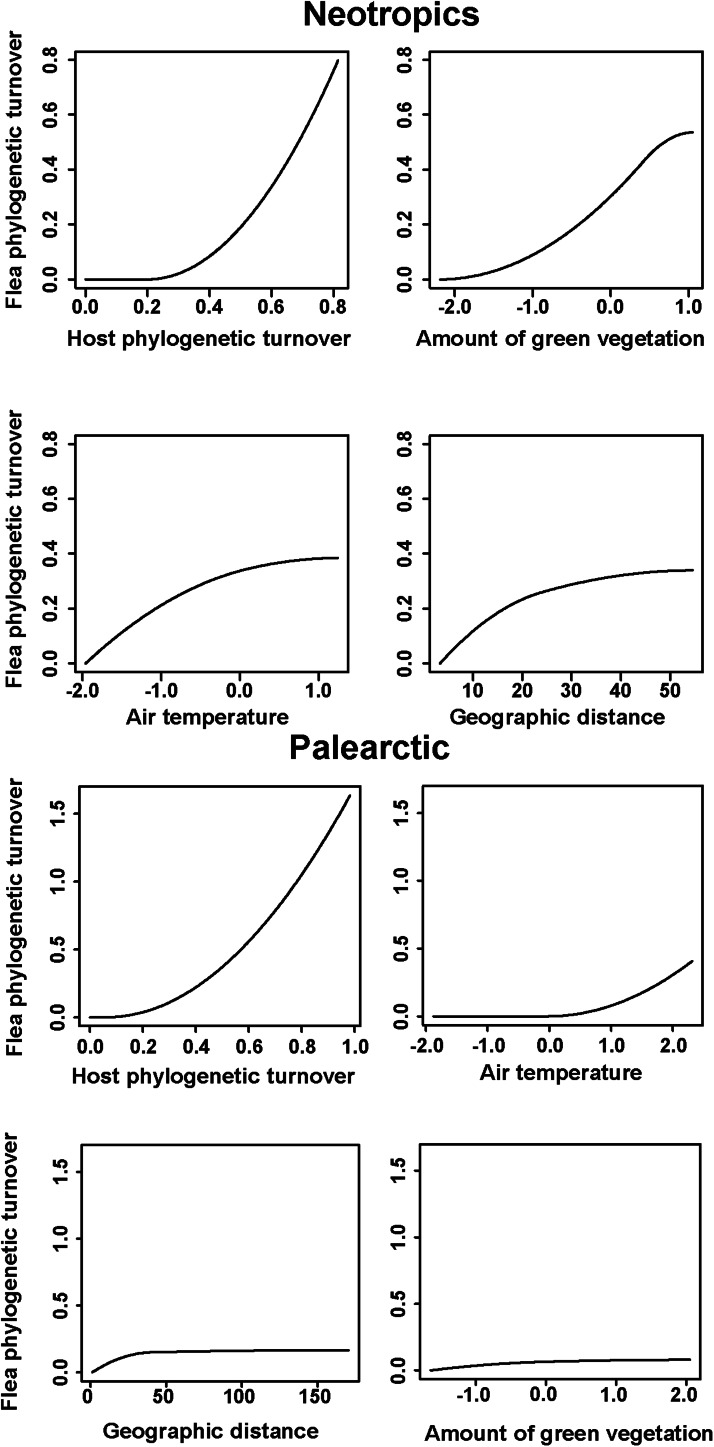
Generalized dissimilarity model-fitted I-splines (partial regression fits) of host phylogenetic turnover, environmental variables and geographic distance as predictors of flea phylogenetic turnover across the Neotropics and the Palaearctic. The steeper slope of the transformed relationship on a given section of the gradient indicates a greater rate of turnover.

The results of the phyloGDMs that aimed to test (a) how flea phylogenetic turnover was affected by host phylogenetic turnover, environmental dissimilarity and geographic distance and (b) how host phylogenetic turnover was affected by environmental dissimilarity and geographic distance are presented in Tables 1 and 2, respectively. All models explained substantial proportions of deviance. I-splines produced by the phyloGDMs for fleas are presented in Figs 1–3, whereas those for hosts are in Supplementary Figs S1–S3. In all realms, flea phylogenetic turnover was affected first and foremost by host phylogenetic turnover, whereas the effect of environment on flea phylogenetic turnover within a realm was weaker. The shape of the I-spline for host turnover as a predictor of flea turnover was similar in all realms and steeply increased with an increase in host phylogenetic dissimilarity. Furthermore, different environmental variables played different roles in their effect on flea phylogenetic turnover. For example, the most important environmental predictor of flea phylogenetic turnover in the Afrotropics, the Indomalaya and the Palaearctic was temperature gradient (Figs 1–3), whereas this factor did not play any role whatsoever in the Australasia and exerted the weakest impact of all the environmental gradients on flea turnover in the Nearctic and the Neotropics (Figs 2 and 3). Similarly, flea phylogenetic turnover strongly responded to the amount of green vegetation gradient in the Neotropics, but the effect of vegetation in the Palaearctic was extremely weak. The precipitation gradient was associated with flea turnover in the Afrotropics, the Australasia and the Nearctic, whereas it did not have any influence in the remaining realms. In addition, dissimilarity in the phylogenetic composition of flea assemblages between regions increased with an increase of spatial distance between regions in all realms, although this increase was manifested differently in different realms, being strong in the Australasia, moderate in the Nearctic and the Neotropics and weak in the Afrotropics, the Indomalaya and the Palaearctic (Figs 1–3).

**Table 1.** Flea phylogenetic turnover as explained by host phylogenetic turnover (HPT), environmental variables (Veg, T, P) and geographic distance (GD) between regions in 6 biogeographic realms

Realm	%Deviance explained	Predictor	I-spline 1	I-spline 2	I-spline 3	Σ_I-splines_
Afrotropics	81.28	HPT	0.00	0.16	0.97	1.97
		Veg	0.00	0.00	0.00	0.00
		T	0.17	0.04	0.03	0.24
		P	0.05	0.07	0.005	0.12
		GD	0.11	0.00	0.00	0.11
Australasia	58.72	HPT	0.00	0.00	1.56	1.56
		Veg	0.00	0.00	0.00	0.00
		T	0.00	0.00	0.00	0.00
		P	0.12	0.00	0.00	0.12
		GD	0.00	0.15	0.55	0.70
Indomalaya	77.71	HPT	0.00	0.00	0.70	0.70
		Veg	0.00	0.00	0.32	0.32
		T	0.32	0.00	0.00	0.32
		P	0.00	0.00	0.00	0.00
		GD	0.00	0.12	0.00	0.12
Nearctic	81.55	HPT	0.00	0.00	1.13	1.13
		Veg	0.00	0.00	0.00	0.00
		T	0.00	0.03	0.08	0.11
		P	0.02	0.00	0.16	0.18
		GD	0.00	0.25	0.00	0.25
Neotropics	75.74	HPT	0.00	0.00	0.81	0.81
		Veg	0.00	0.54	0.00	0.54
		T	0.27	0.11	0.00	0.38
		P	0.00	0.00	0.00	0.00
		GD	0.20	0.13	0.00	0.33
Palaearctic	74.47	HPT	0.00	0.00	1.65	1.65
		Veg	0.05	0.03	0.00	0.08
		T	0.00	0.00	0.42	0.42
		P	0.00	0.00	0.00	0.00
		GD	0.15	0.02	0.00	0.17

Veg, T and P composite environmental variables reflecting the amount of green vegetation, air temperature and precipitation, respectively (see text for explanations); I-splines 1, 2 and 3: coefficients of the first, second or third I-spline, respectively; Σ_I-splines_: sum of 3 I-splines (demonstrates the amplitude of an I-spline).

**Table 2.** Host phylogenetic turnover as explained by environmental variables (Veg, T, P) and geographic distance (GD) between regions in 6 biogeographic realms

Realm	Deviance explained	Predictor	I-spline 1	I-spline 2	I-spline 3	Σ_I-splines_
Afrotropics	19.08	Veg	0.00	0.00	0.00	0.00
		T	0.00	0.00	0.13	0.13
		P	0.00	0.91	0.00	0.91
		GD	0.00	0.00	0.00	0.00
Australasia	38.61	Veg	0.06	0.17	0.00	0.23
		T	0.00	0.00	0.09	0.09
		P	0.00	0.00	0.00	0.00
		GD	0.37	0.00	0.21	0.58
Indomalaya	53.81	Veg	0.08	0.00	0.00	0.08
		T	0.00	0.60	0.00	0.60
		P	0.03	0.00	0.00	0.03
		GD	0.00	0.22	0.00	0.22
Nearctic	52.57	Veg	0.00	0.00	0.00	0.00
		T	0.00	0.00	0.35	0.35
		P	0.07	0.00	0.12	0.19
		GD	0.47	0.32	0.00	0.79
Neotropics	67.31	Veg	0.02	0.06	0.00	0.08
		T	0.08	0.24	0.00	0.32
		P	0.00	0.00	0.04	0.04
		GD	0.15	0.40	0.04	0.58
Palaearctic		Veg	0.04	0.00	0.00	0.04
		T	0.15	0.00	0.26	0.41
		P	0.54	0.00	0.11	0.65
		GD	0.33	0.41	0.26	1.00

Veg, T and P composite environmental variables reflecting the amount of green vegetation, air temperature and precipitation, respectively (see text for explanations); I-splines 1, 2 and 3: coefficients of the first, second or third I-spline, respectively; Σ_I-splines_: sum of 3 I-splines (demonstrates the amplitude of an I-spline).

Within the same realm, dissimilarity in host and flea phylogenetic composition was differently affected by environmental gradients. For example, the effect of precipitation on dissimilarity in phylogenetic composition in the Afrotropics was strong for hosts and weak for fleas (Fig. 1 and Supplementary Fig. S1). In the Australasia, the amount of vegetation was a strong predictor of host, but not flea, phylogenetic turnover (Fig. 1 and Supplementary Fig. S1). In 4 of 6 realms (the Australasia, the Nearctic, the Neotropics and the Palaearctic) (Figs 1–3 and Supplementary Figs S1–S3), geographic distances were substantially better predictors of host phylogenetic turnover than environmental gradients.

### Phylogenetic originality, host specificity and phylogenetic diversity of flea assemblages

**Fig. 4.**
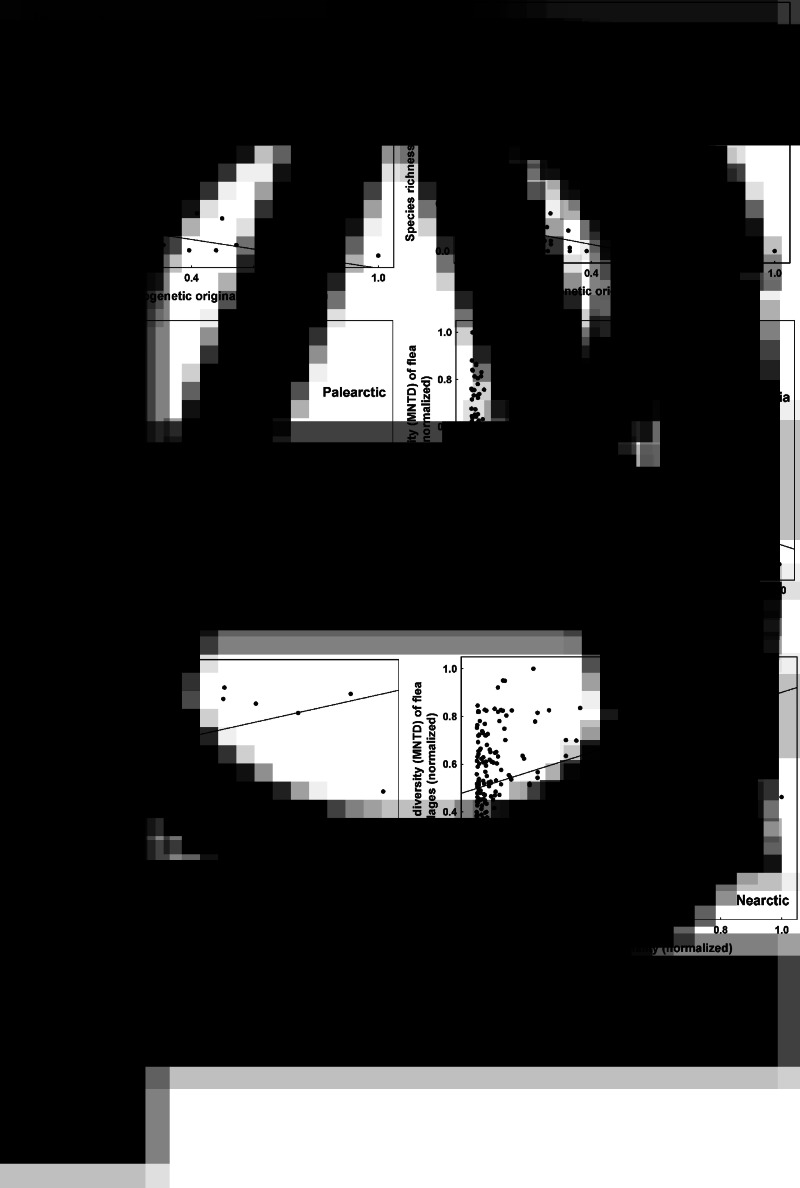
Relationship between the phylogenetic originality of a flea species and the number of host species it exploits in the Palaearctic (A), between the phylogenetic originality of a host species and the number of flea species it harbours in the Afrotropics (B) and the Palaearctic (C), between the phylogenetic originality of a host species and the phylogenetic diversity of its flea assemblage measured as either MPD or MNTD in the Australasia (D) and the Nearctic (E, F). MPD, mean pairwise distance; MNTD, mean nearest-taxon distance (see text for explanations).

No significant relationships between a flea species’ phylogenetic originality (FPO) and either its host spectrum's size (HSR) or phylogenetic diversity were found except in the Palaearctic (*P* > 0.05 for all), where the number of host species exploited by a flea decreased with an increase in a flea's originality (HSR = 0.15–0.20 × FPO, *R*^2^ = 0.02, *F* = 4.89, *P* = 0.03; Fig. 4A). However, the coefficient of determination of this relationship was rather low, and the point scatter in Fig. 4A was clearly triangular, suggesting that fleas with a low degree of originality could exploit either many or only a few host species, whereas highly original fleas were highly host specific. In addition, the negative relationship between the FPO and the phylogenetic diversity of its host spectrum, in terms of MPD, was marginally significant in the Nearctic (*F* = 3.10, *P* = 0.08).

A host's phylogenetic originality (HPO) affected the species richness of its flea assemblage in the Afrotropics and the Palaearctic, both metrics of phylogenetic diversity in the Nearctic, and phylogenetic diversity measured as MNTD in the Australasia (Table 3), whereas no relationships whatsoever were found in the Neotropics and the Indomalaya. In the Afrotropics, the Palaearctic and the Australasia, these relationships were negative, and similar to the effect of FPO on host diversity, point scatters were clearly triangular (Fig. 4B–D). This suggested that phylogenetically non-original host species could harbour either highly diverse or less diverse flea assemblages, whereas flea assemblages of highly phylogenetically original hosts were either species-poor or phylogenetically similar. However, in the Nearctic, both the MPD and MNTD of flea assemblages increased with an increase in HPO (Fig. 4E, F). In other words, flea assemblages of highly phylogenetically original North American hosts were highly phylogenetically diverse. A significantly negative relationship between MNTD and HPO in the Australasia arose mainly due to 2 monotreme hosts (which obviously were highly phylogenetically original) (Fig. 4D). After these hosts were removed from the analysis, the relationship still appeared to be negative, albeit only marginally significant (MNTD = 0.55–1.77 × HFO, *F* = 3.56, *P* = 0.06). Additionally, negative relationships between HPO and (i) species richness or (ii) phylogenetic dissimilarity, measured as the MPD of its flea assemblage, were marginally significant in the Nearctic (*F* = 3.10, *P* = 0.08) and the Australasia (*F* = 3.80, *P* = 0.06), respectively.

**Table 3.** Relationship between the phylogenetic originality of a host species (HPO) and either the species richness (FSR) or phylogenetic diversity of its flea assemblage (MPD or MNTD) in 4 biogeographic realms (only significant models are shown)

Realm	Diversity metric	Equation	*R^2^*	*F*	*P*
Afrotropics	FSR	0.13 − 0.24 × HPO	0.06	9.22	0.003
Palaearctic	FSR	0.14 − 0.20 × HPO	0.04	7.54	0.007
Australasia	MNTD	0.52 − 0.42 × HPO	0.07	7.35	0.008
Nearctic	MPD	0.62 + 0.29 × HPO	0.04	7.45	0.006
	MNTD	0.50 + 0.40 × HPO	0.08	15.27	0.0001

MPD, mean pairwise distance; MNTD, mean nearest-taxon distance (Tucker *et al*., 2017).

## Discussion

The strong link between flea and host phylogenetic turnover found in this study reflects the common evolutionary history of fleas and their hosts. This common history does not, however, necessarily imply co-speciation as advocated in the earliest studies that compared parasite and host phylogenies (e.g. Hafner and Nadler, 1988, 1990; Hafner and Page, 1995). Instead, the common history of parasites and their hosts is often (indeed, almost always) characterized by a variety of other coevolutionary events, such as host switching, lineage sorting and duplications [see definitions in, e.g. Paterson *et al*. (1993); Beveridge and Chilton (2001); Roy (2001)]. As a result, phylogenetic trees of parasites and hosts are most often incongruent (e.g. Caira and Jensen, 2001). Our results indicated a pattern of parasite–host coevolution resembling co-speciation but taking place at a phylogenetic level deeper than species and genera. This supports the conclusions of Traub (1980), Whiting *et al*. (2008) and Zhu *et al*. (2015) that flea history and dispersal are strongly tied to those of their mammalian hosts and that flea diversification started when their ancestors became an ectoparasite on mammals. Moreover, flea diversification is undoubtedly a response to the diversification of their hosts (e.g. Morrone and Gutiérrez, 2005) exemplifying the so-called phylogenetic tracking (Russo *et al*., 2017). Although strict co-speciation has not been proven, repeated episodes of co-speciation might be masked because they are mediated by, for example, vicariant events. Clear clustering of flea assemblages, according to the biogeographic realms to which they belong (Krasnov *et al*., 2022*a*), indicated independent (to some extent) flea evolution in different realms. Tight relationships of certain phylogenetic lineages of fleas with certain phylogenetic lineages of their hosts (e.g. Krasnov *et al*., 2016) have resulted in patterns of flea geographic distribution that often mirror those of their host (Traub, 1980; Medvedev, 2005; Morrone and Gutiérrez, 2005; López-Berrizbeitia *et al*., 2020). Recently, Gibert *et al*. (2021) demonstrated that the species composition of fleas and their small mammalian hosts on several continents predominantly depended on historical processes (dispersal). This is because fleas most likely rely on their hosts, using them as dispersal vehicles due to their own limited (if any) dispersal abilities.

Environmental predictors of flea phylogenetic turnover played a less important, albeit substantial, role than host phylogenetic turnover. This indicates the dependence of a flea's ecological requirements on both host identity and environmental factors (Krasnov *et al*., 1998, 2015) and could be a reasonable explanation for the effect of environment on flea compositional turnover. The environmental effect on phylogenetic turnover suggests that some physiological flea traits determining their environmental requirements and/or preferences might be phylogenetically conserved. This, however, has never been studied and, thus, is completely unknown. Nevertheless, some observations have indicated that this might well be the case. For example, a significant phylogenetic signal was found in flea body size (Surkova *et al*., 2018). Given that body size and many physiological variables are highly correlated in many taxa, including insects (Peters, 1983; Schmidt-Nielsen, 1984; Chown and Gaston, 1997; Chown *et al*., 2002), a phylogenetic signal in flea physiological traits is expected, which, in turn, might be translated into the relationships between flea phylogenetic turnover and environmental gradients. Furthermore, environmental predictors of flea phylogenetic turnover differed between biogeographic realms. There might be at least 2, not mutually exclusive, reasons behind this. First, the length of an environmental gradient, that is, variation in an environmental variable, can differ between realms, so that environmental variation might be too low for a turnover to respond to. For example, the coefficients of variation of the NDVI variables in the Australasia ranged from 0.47 to 0.52, whereas the coefficients of variation of the precipitation variables were higher and ranged from 0.93 to 1.11. As a result, flea phylogenetic turnover responded to precipitation but not to the amount of green vegetation. Second, different flea lineages might have different environmental requirements/preferences in dependence on the environmental conditions they evolved under, so that lineages in different realms differ in their sensitivity to the same environmental factors. Admittedly, we do not have any information supporting the latter explanation. We recognize that it is highly speculative and requires special investigation.

As mentioned above, host phylogenetic turnover appeared to be a better predictor of flea phylogenetic turnover than environment. A similar pattern was reported by Krasnov *et al*. (2020) for flea compositional turnover in 4 biogeographic realms. However, the best predictor of dissimilarity in flea species composition in Mongolia was found to be the air temperature gradient, whereas the effect of dissimilarity in host species composition was weaker (Maestri *et al*., 2017). In other words, the identity of the best predictor of flea compositional turnover might be scale dependent. Comparison of this study's results with those of Krasnov *et al*. (2019*b*) and Maestri *et al*. (2020), however, suggests that predictors of flea phylogenetic turnover are scale invariant. Nevertheless, both studies at the local scale were carried out in the Palaearctic. It remains to be further studied whether predictors of flea phylogenetic turnover are scale-dependent in other biogeographic realms.

Phylogenetic turnovers of fleas and hosts were predicted by different environmental gradients in the same realm. Maestri *et al*. (2017, 2020) reported the same pattern for the compositional beta-diversity of fleas and hosts and concluded that flea species composition responds directly to environmental variables rather than being mediated by host responses. The same appears to be true for the phylogenetic beta-diversity of fleas. The simplest explanation for this may be the sharp differences in ecological requirements, possibilities and constraints between insects (i.e. fleas) and mammals.

Geographic distance was, in general, a better predictor of phylogenetic turnover for hosts than for fleas. Its importance varied between realms in both fleas and hosts. In addition, it had no effect on the phylogenetic turnover of hosts in the Afrotropics. This supports earlier observations that the famous pattern of distance decay of community similarity (Nekola and White, 1999) is not universal in terms of either compositional similarity or phylogenetic similarity (Pérez-del-Olmo *et al*., 2009; Maestri *et al*., 2017).

We found only a weak relationship between flea phylogenetic originality and host specificity and only in terms of host species richness and only in the Palaearctic (although some trend could be envisaged in the Nearctic). The main reason behind the general lack of association between parasite phylogenetic originality and host specificity could be that host specificity, to a great extent, correlates with parasite traits rather than with their phylogenetic position. For example, reproductive output, in terms of clutch size, correlates with host specificity in copepods (Doherty *et al*., 2022). Similarly, the number of host species used by facultatively haematophagous gamasid mites correlated with their body size (Krasnov *et al*., 2013). In other words, parasite host specificity might be related to its functional rather than its phylogenetic originality, while phylogenetic originality does not necessarily reflect functional originality (Pavoine *et al*., 2017). The relationships between a parasite's functional originality and its host specificity remain to be investigated. Nevertheless, the case of the Palaearctic fleas may be linked to the fact that diversification of the largest flea families likely occurred in this realm and started as late as in the Eocene (Medvedev, 2005). As a result, the distribution of flea species according to the degree of their phylogenetic originality was strongly asymmetrical in the Palaearctic (skewness = 3.94) followed by that in the Nearctic (3.38), with less asymmetry in the remaining realms (2.33–3.24). The high asymmetry of this distribution could have resulted in a significant relationship between phylogenetic originality and the size of a host spectrum.

Relationships between the phylogenetic originality of a host species and either the compositional or phylogenetic diversity of their flea assemblages, albeit weak, were found in 4 of the 6 realms. These might have resulted from the aforementioned chain of associations between phylogenetic originality, geographic range size and diversity of flea assemblages (Feliu *et al*., 1997; Veron *et al*., 2021). Surprisingly, the trend of increased phylogenetic flea diversity in the most phylogenetically original hosts in the Nearctic was opposite to the patterns found in the Australasia. This difference is difficult to explain, although it suggests differential histories of flea evolution in different realms. Our knowledge of the historical patterns of flea evolution and dispersal is insufficient. However, these opposite trends might somehow be associated with the fact that flea fauna in the Nearctic is represented by a large number of species belonging to the youngest family (Ceratophyllidae), whereas many Australasian fleas belong to the basal flea families Macropsyllidae, Stephanocircidae, Pygiopsyllidae, Stivaliidae and Lycopsyllidae (Medvedev, 2005; Zhu *et al*., 2015).

In conclusion, the results of our study suggest that fleas’ phylogenetic turnover is determined, to a great extent, by the phylogenetic turnover of their hosts, whereas environmental factors are less important. Comparison of the results of this study with those of earlier studies (e.g. Krasnov *et al*., 2019*a*) suggests that the phylogenetic alpha- and beta-diversity of parasites are controlled by different rules (see also Krasnov *et al*., 2019*b*). In addition, phylogenetic patterns in the order Siphonaptera are manifested mainly at the level of regional assemblages rather than at the level of individual species.

## Data Availability

Data for the Afrotropics, the Neotropics, the Nearctic and the Palaearctic are deposited in the Mendeley Data Repository (http://dx.doi.org/10.17632/6jxpf678rm.1) (Krasnov, 2018). Data for the Australasia and the Indomalaya data can be found in the sources cited in Krasnov *et al*. (2022*a*).

## Supplementary material

For supplementary material accompanying this paper visit https://doi.org/10.1017/S003118202300015X. click here to view supplementary material
