# Supplementary material for: Phylogenetic patterns in regional flea assemblages from 6 biogeographic realms: strong links between flea and host phylogenetic turnovers and weak effects of phylogenetic originality on host specificity
Source: Parasitology. 2023 Feb 17;150(5):455–67. doi: 10.1017/S003118202300015X (PMC10089810; doi:10.1017/S003118202300015X)
Supplement: Supplementary file 1 [file S003118202300015Xsup001.docx]

**Supplementary table.**

**Table S1.** Summary of principal component analyses of environmental variables in six biogeographic realms.

| Realm |  |  | Veg |  |  | P |  |  | T |  |
| --- | --- | --- | --- | --- | --- | --- | --- | --- | --- | --- |
|  |  | E | PV | R | E | PV | R | E | PV | R |
| Afrotropics |  | 3.59 | 0.90 |  | 2.12 | 0.71 |  | 2.56 | 0.74 |  |
|  | NDVIa |  |  | 0.96 |  |  |  |  |  |  |
|  | NDVIsp |  |  | 0.91 |  |  |  |  |  |  |
|  | NDVIsu |  |  | 0.96 |  |  |  |  |  |  |
|  | NDVIw |  |  | 0.95 |  |  |  |  |  |  |
|  | Pa |  |  |  |  |  | 0.91 |  |  |  |
|  | Psp |  |  |  |  |  | 0.92 |  |  |  |
|  | Psu |  |  |  |  |  | 0.48 |  |  |  |
|  | Pw |  |  |  |  |  | 0.81 |  |  |  |
|  | Tmax |  |  |  |  |  |  |  |  | 0.61 |
|  | Tmean |  |  |  |  |  |  |  |  | 0.96 |
|  | Tmin |  |  |  |  |  |  |  |  | 0.91 |
| Australasia |  | 3.90 | 0.97 |  | 3.69 | 0.92 |  | 2.92 | 0.97 |  |
|  | NDVIa |  |  | 0.98 |  |  |  |  |  |  |
|  | NDVIsp |  |  | 0.99 |  |  |  |  |  |  |
|  | NDVIsu |  |  | 0.99 |  |  |  |  |  |  |
|  | NDVIw |  |  | 0.99 |  |  |  |  |  |  |
|  | Pa |  |  |  |  |  | 0.99 |  |  |  |
|  | Psp |  |  |  |  |  | 1.00 |  |  |  |
|  | Psu |  |  |  |  |  | 0.94 |  |  |  |
|  | Pw |  |  |  |  |  | 0.91 |  |  |  |
|  | Tmax |  |  |  |  |  |  |  |  | 0.98 |
|  | Tmean |  |  |  |  |  |  |  |  | 1.00 |
|  | Tmin |  |  |  |  |  |  |  |  | 0.98 |
| Indomalaya |  | 3.15 | 0.79 |  | 3.02 | 0.76 |  |  |  |  |
|  | NDVIa |  |  | 0.77 |  |  |  | 2.67 | 0.89 |  |
|  | NDVIsp |  |  | 0.93 |  |  |  |  |  |  |
|  | NDVIsu |  |  | 0.92 |  |  |  |  |  |  |
|  | NDVIw |  |  | 0.91 |  |  |  |  |  |  |
|  | Pa |  |  |  |  |  | 0.74 |  |  |  |
|  | Psp |  |  |  |  |  | 0.97 |  |  |  |
|  | Psu |  |  |  |  |  | -0.77 |  |  |  |
|  | Pw |  |  |  |  |  | 0.97 |  |  |  |
|  | Tmax |  |  |  |  |  |  |  |  | 0.88 |
|  | Tmean |  |  |  |  |  |  |  |  | 0.99 |
|  | Tmin |  |  |  |  |  |  |  |  | 0.95 |
| Nearctic |  | 3.21 | 0.80 |  | 3.06 | 0.77 |  | 2.86 | 0.95 |  |
|  | NDVIa |  |  | 0.99 |  |  |  |  |  |  |
|  | NDVIsp |  |  | 0.97 |  |  |  |  |  |  |
|  | NDVIsu |  |  | 0.75 |  |  |  |  |  |  |
|  | NDVIw |  |  | 0.86 |  |  |  |  |  |  |
|  | Pa |  |  |  |  | 0.96 |  |  |  |  |
|  | Psp |  |  |  |  | 0.95 |  |  |  |  |
|  | Psu |  |  |  |  | 0.78 |  |  |  |  |
|  | Pw |  |  |  |  | 0.80 |  |  |  |  |
|  | Tmax |  |  |  |  |  |  |  |  | 0.96 |
|  | Tmean |  |  |  |  |  |  |  |  | 1.00 |
|  | Tmin |  |  |  |  |  |  |  |  | 0.97 |
| Neotropics |  | 3.75 | 0.94 |  | 3.09 | 0.77 |  | 2.51 | 0.84 |  |
|  | NDVIa |  |  | 0.98 |  |  |  |  |  |  |
|  | NDVIsp |  |  | 0.97 |  |  |  |  |  |  |
|  | NDVIsu |  |  | 0.97 |  |  |  |  |  |  |
|  | NDVIw |  |  | 0.96 |  |  |  |  |  |  |
|  | Pa |  |  |  |  |  | 0.94 |  |  |  |
|  | Psp |  |  |  |  |  | 0.95 |  |  |  |
|  | Psu |  |  |  |  |  | 0.90 |  |  |  |
|  | Pw |  |  |  |  |  | 0.70 |  |  | 0.81 |
|  | Tmax |  |  |  |  |  |  |  |  | 0.98 |
|  | Tmean |  |  |  |  |  |  |  |  | 0.94 |
|  | Tmin |  |  |  |  |  |  |  |  |  |
| Palearctic |  | 2.89 | 0.72 |  | 3.07 | 0.77 |  | 2.59 | 0.86 |  |
|  | NDVIa |  |  | 0.97 |  |  |  |  |  |  |
|  | NDVIsp |  |  | 0.95 |  |  |  |  |  |  |
|  | NDVIsu |  |  | 0.62 |  |  |  |  |  |  |
|  | NDVIw |  |  | 0.81 |  |  |  |  |  |  |
|  | Pa |  |  |  |  |  | 0.97 |  |  |  |
|  | Psp |  |  |  |  |  | 0.94 |  |  |  |
|  | Psu |  |  |  |  |  | 0.75 |  |  |  |
|  | Pw |  |  |  |  |  | 0.83 |  |  |  |
|  | Tmax |  |  |  |  |  |  |  |  | 0.87 |
|  | Tmean |  |  |  |  |  |  |  |  | 1.00 |
|  | Tmin |  |  |  |  |  |  |  |  | 0.92 |

Veg, P, and T: the first principal components of NDVI (Normalized Difference Vegetation Index), precipitation, and air temperature, respectively; E: eigenvalue; PV: proportion of variation explained; R: linear correlation between a principal component and an original environmental variable; NDVIa, NDVIsp, NDVIsu, and NDVIw: Normalized Difference Vegetation Indices for (boreal or austral) autumn, spring, summer, and winter, respectively; Pa, Psp, Psu, and Pw: mean precipitation in autumn, spring, summer, and winter, respectively; Tmax, Tmin, and Tmean: maximal, minimal, and mean annual air temperature, respectively.

**Supplementary figures**

**Fig. S1.** Generalized dissimilarity model-fitted I-splines of environmental variables and geographic distance as predictors of host phylogenetic turnover in the Afrotropics and the Australasia.





**Fig. S2.** Generalized dissimilarity model-fitted I-splines of environmental variables and geographic distance as predictors of host phylogenetic turnover in the Indomalaya and the Neotropics.





**Fig. S3.** Generalized dissimilarity model-fitted I-splines of environmental variables and geographic distance as predictors of host phylogenetic turnover in the Neotropics and the Palearctic.
